# Change in Oral Health-Related Quality of Life Following Minimally Invasive Aesthetic Treatment for Children with Molar Incisor Hypomineralisation: A Prospective Study

**DOI:** 10.3390/dj6040061

**Published:** 2018-11-01

**Authors:** Noren Hasmun, Jennifer Lawson, Mario V. Vettore, Claire Elcock, Halla Zaitoun, Helen Rodd

**Affiliations:** 1 School of Clinical Dentistry, University of Sheffield, Sheffield S10 2TA, UK; m.vettore@sheffield.ac.uk (M.V.V.); c.elcock@sheffield.ac.uk (C.E.); h.d.rodd@sheffield.ac.uk (H.R.); 2 Department of Paediatric Dentistry, Charles Clifford Dental Hospital, Sheffield S10 2SZ, UK; Jennifer.Lawson@sth.nhs.uk (J.L.); Halla.Zaitoun@sth.nhs.uk (H.Z.)

**Keywords:** oral health-related quality of life, children, molar incisor hypomineralisation, incisor opacities, minimally invasive treatment

## Abstract

Molar incisor hypomineralisation (MIH) is a common enamel condition, presenting with incisor opacities, which may be of psychosocial concern to children. This clinical study sought to determine whether minimally invasive treatment, aiming to improve incisor aesthetics, would also improve children’s oral health-related quality of life (OHRQoL). 111 MIH patients, aged 7–16 years, referred to a UK Dental Hospital, were invited to complete the Child Oral Health Impact Profile (C-OHIP-SF19) prior to any intervention (T_0_) and again at one-month following the intervention (T_1_) for MIH. Treatment regimens included one or more of the following: Microabrasion; resin infiltration; tooth whitening; resin composite restoration. Data were obtained for 93 children with a mean age of 11 years. Mean total C-OHIP-SF19 score at T_0_ was 47.00 (SD = 9.29; range = 0–76) and this increased significantly at T_1_ to 58.24 (SD = 9.42; range = 0–76; *p* < 0.001, paired *t*-test), indicating a marked improvement in self-reported OHRQoL. There were no statistically significant differences according to gender. This is the first study to show that simple, minimally invasive dental treatment, to reduce the visibility of enamel opacities, in MIH, can have a positive impact on children’s wellbeing.

## 1. Introduction

It is well recognised that children with developmental enamel defects may experience a range of psychosocial impacts [1]. For young people with visible defects, poor dental appearance and a sense of feeling different may have a particularly negative effect on social interactions and self-esteem [2,3]. However, the reported severity and nature of these impacts can be hugely variable, depending on the child’s sense of self and the social context at different time points in their life, such as moving to a new school [4,5]. It has also been suggested that children with highly visible incisor opacities may be exposed to negative social judgements from their peers, with the common misconception that affected individuals do not care about their appearance or are lazy about brushing their teeth [6].

The first studies to consider the potential impact of abnormal dental development, in terms of poor dental aesthetics, focused on dental fluorosis. Children, living in a number of African countries with endemic dental fluorosis, frequently reported a reluctance to smile, as they did not like to show their discoloured teeth [7]. Investigators have also explored the psychosocial impact of amelogenesis imperfecta in children and young adults using both quantitative and qualitative approaches [2,3,8]. This genetic condition presents both aesthetic and functional problems for individuals, as well as a lifelong treatment burden. For some, the negative impacts of having poor dental appearance are overwhelming, to the detriment of many facets of normal social interaction, such as meeting new people or forming relationships. Recently, attention has turned to a more common enamel disorder, molar incisor hypomineralisation (MIH), which may also severely compromise dental appearance and function. An estimated 13–14% of children are thought to have MIH, creating immense challenges for oral health providers across the world [9,10]. In addition to poorly mineralised and sensitive first permanent molars, MIH typically presents with one or more affected permanent incisors in a reported third of cases, which may have a variety of discrete yellow/white/cream/brown opacities involving the incisal third of labial surfaces [11]. However, in contrast to affected permanent molars, the incisors tend not to be as sensitive or suffer from post-eruptive tissue breakdown. The first studies to explore the psychosocial impacts of MIH were conducted with Brazilian children, using the Child Perception Questionnaire (CPQ11-14ISF) [12] and a questionnaire on tooth appearance, which had originally been validated for use with children with fluorosis [13]. Findings from both these studies suggested that children with severe MIH were dissatisfied with tooth colour, avoided smiling, and experienced poorer social well-being.

Anecdotal reports from paediatric dentists suggest that they are seeing increasing numbers of children with MIH, sometimes as young as six or seven years of age, who report being upset by ‘marks’ on their teeth and who request ‘corrective’ treatment. In these situations, clinicians should discuss the risks/benefits of any dental intervention whilst being empathetic to the child’s treatment expectations and concerns. It is also imperative that minimally invasive approaches are adopted to preserve tooth structure and maintain tooth vitality, whilst reducing the visibility of any opacity as much as possible. A variety of treatment regimens have been described for the management of children with hypomineralised permanent anterior teeth, including: Tooth whitening (using hydrogen peroxide-containing products); microabrasion; resin infiltration; and resin composite restoration [14,15,16]. Although guidance has been published by the European Academy of Paediatric Dentistry for improving incisor aesthetics in children with MIH [17], the evidence-base for the effectiveness of any single or combined treatment regimen is completely lacking, leaving clinicians to take a pragmatic and experience-led approach.

Although there is persuasive evidence that children with enamel defects experience a variety of psychosocial impacts, surprisingly few studies have attempted to determine the value of dental treatment in addressing these negative impacts in young patients. To date, only two studies, using non-validated questionnaires, have sought children’s perspectives of aesthetic interventions to manage their visible incisor opacities [18,19]. There is thus great scope to undertake this line of enquiry, given the prevalence of enamel defects, the negative impacts they may have, and the recognition that seeking patient-reported outcomes and experiences is integral to high quality service provision and development [20].

The aim of this study, therefore, was to compare OHRQoL in children with MIH before and after minimally invasive dental treatment to reduce the visibility of their incisor opacities. An additional objective was to consider whether there was any difference in reported OHRQoL before and after dental treatment according to gender and age.

## 2. Materials and Methods

### 2.1. Overall Study Design

This was a prospective clinical intervention study conducted in a public-funded dental hospital in Sheffield, UK. It formed part of a more comprehensive three-year PhD study, undertaken by the lead author (N.H.). Children with MIH, requesting cosmetic improvement of one or more permanent anterior teeth, were offered a range of minimally invasive treatment regimens. Prior to treatment they completed a validated OHRQoL measure, and this was repeated at their one-month ‘post treatment’ review. Children were recalled at six months following treatment; data collection is ongoing and will be published in a future communication.

### 2.2. Recruitment of Participants

Ethical approval was granted for this study by the UK National Research Ethics Committee (Ref. 17/WA/0096). Participants were sought from the paediatric dentistry clinic of a British dental hospital, following referral from their general dental practitioner for specialist management of their MIH. Recruitment and subsequent treatment took place between June 2017 and July 2018, and was undertaken by two of the named authors (N.H. and J.L.). Inclusion and exclusion criteria were as follows.

Inclusion criteria:

- Children diagnosed with MIH, according to well established clinical criteria [21] and confirmed by a consultant in paediatric dentistry (H.R.);
- children with MIH and who had a visible enamel opacity involving at least one permanent incisor;
- children who requested improvement in their incisor aesthetics; and
- children aged between 7 and 16 years.

Exclusion criteria:

- Children who presented with an acute dental symptom and required urgent treatment;
- children who were planned to undergo active treatment for their hypomineralised molars during the proposed study period (e.g., extractions) or orthodontic treatment;
- children with any dental or facial anomaly other than MIH (e.g., hypodontia, cleft lip, and palate);
- children with compromised incisor aesthetics due to a traumatic dental injury, tooth surface loss, or caries;
- children with severe learning disabilities who were unable to understand and take part in the research, even with support from the research team; and
- children or parents who did not speak English.

Following initial attendance those who met the inclusion criteria were given an age-appropriate information sheet, as were their parents/carers. At their subsequent treatment appointment, having had time to reflect, they were invited to sign a written consent form, if they wished to participate in the study. 

### 2.3. Sample Size Calculation

The primary outcome measure was the score derived from the Child Oral Health Impact Profile Short Form 19 questionnaire (C-OHIP-SF19), which is described below [22]. A sample size calculation was carried out with a view to testing for any statistically significant difference between mean C-OHIP-SF19 scores pre- and post-treatment intervention, assuming a mean change (reduction) in C-OHIP score of 2.5 and a standard deviation of 8.0 [23]. It was calculated that a sample size of 85 children would result in a study with 81% power and a 5% level of significance. Furthermore, assuming a dropout rate of 10% between baseline and follow-up data collection and applying the following formula, N1 = n/(1 − d); n (participant required) = 85 and d (dropout rate) = 0.1, a total of 95 participants was sought.

### 2.4. Data Collection and Measures

Prior to any dental treatment participants were invited to complete the C-OHIP-SF19. This is a self-report oral health-related quality of life questionnaire, which measures both positive and negative impacts of oral conditions in children on their overall lives and has been used extensively in children’s oral health research [24]. The short form has 19 items encompassing three domains: Oral health, functional well-being, and socio-emotional well-being, and has been validated for use in 8–15 year olds [22]. Participants are asked to report on the frequency of any impact over the past three months on a 5-point Likert scale, which is scored from 0–4 (never, almost never, sometimes, fairly often, almost all the time) with negative items having their score reversed. In addition, this instrument also has one general question concerning the participant’s perception of their overall oral health. The total score can range from 0–76, with the higher score reflecting better OHRQoL. 

The following demographic and clinical data were obtained for each participant from their clinical records:

- Age;
- gender;
- postcode address: To allow categorisation of the participant’s socioeconomic status according to quintile, where 5 represents the greatest level of deprivation and 1 the least [25]; and
- ethnic group (self-reported by the parent/carer).

**Figure 1:**
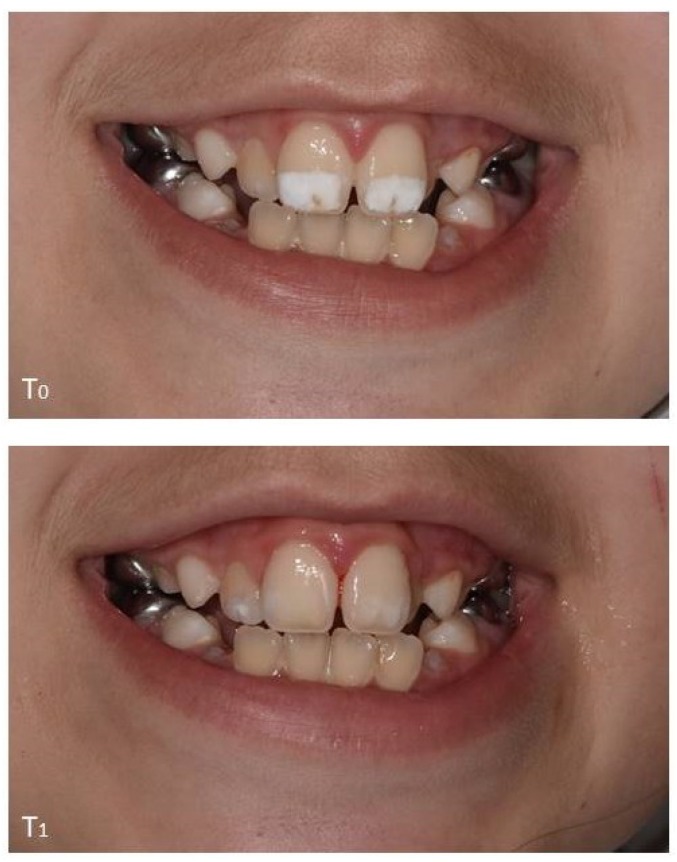
Clinical images to show the appearance of a ‘typical’ participant’s permanent central maxillary incisors before (T_0_) and one-month after (T_1_) treatment (microabrasion followed by resin infiltration).

Standard clinical photographs were acquired of children’s anterior teeth before any treatment at baseline (T_0_) and at one-month review (T_1_) using a digital SLR camera (Nikon D3400, Nikon UK Ltd., Kingston upon Thames, UK) equipped with a Sigma EM 140DG macro ring flash (Sigma Imaging (UK) Ltd., Welwyn Garden City, UK) and Tamron 90 mm macro lens (TAMRON Europe GmbH, Berkshire, UK). These clinical images were taken at standardised settings (ISO 100, 1/160 speed and F/22 aperture), distance (about 20 cm between operator and the patient), and natural and room illumination conditions. Some examples of these are shown in Figure 1. Images were stored electronically as part of the patients’ dental records.

### 2.5. Statistical Analysis

Data were entered using the Statistical Package for Social Sciences (SPSS) v24.0 (IBM Corp., Chicago, IL, USA). Age, gender, ethnicity, deprivation (quintile) score, and MHSI were subject to simple descriptive analysis for means (standard deviations, range) and proportions. C-OHIP-SF19 data distribution were assessed for normality (Shapiro test) and a Wilcoxon Signed Rank test was used to determine any significant difference between pre- and post-treatment scores with a significance level set at 5% (*p* ≤ 0.05). Differences in C-OHIP-SF19 scores according to gender and age groups were determined using a Mann-Whitney U test. It was planned that for any missing data in the C-OHIP-SF19 questionnaires, the median score for each item would be imputed. Questionnaires with more than 50% of missing responses would be excluded from analysis.

### 2.6. Clinical Intervention

**Figure 2:**
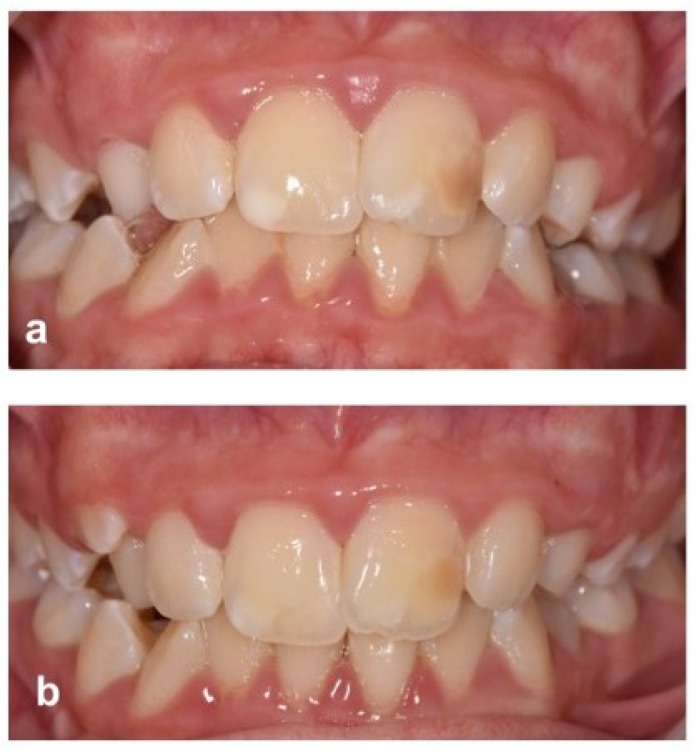
Clinical images to demonstrate both a successful clinical outcome and unacceptable aesthetic result. (**a**) Maxillary central incisors showing cream/white opacity (right) and white and brown opacities (left) pre-treatment. (**b**) Microabrasion followed by resin infiltration achieved good aesthetic results on the right central incisor, but brown opacity was still visible on the left central incisor, necessitating further resin composite restoration.

Children presented with a variety of white, cream, yellow, and brown opacities involving any of their maxillary and/or mandibular permanent incisors, and occasionally also involving their canines. These areas of hypomineralisation were generally discrete and well defined, affecting the incisal third of labial/buccal surfaces. Post-eruptive tooth tissue loss/hypoplasia was uncommon. Due to the diversity of lesion presentation, treatment regimens were tailored for individual patients on discussion with the lead consultant (H.R.), together with the child and their parent/carers. However, the most common approach was for an initial two to three cycles of microabrasion (Opalustre^TM^, Optident Ltd., Ilkley, UK), immediately followed by resin infiltration (ICON^TM^, DMG, Hamburg, Germany). Some children had microabrasion alone, and others had ICON alone. For children with multiple affected teeth, a tooth whitening gel (Opalescence^TM^ 16% carbamide peroxide, Optident Ltd., Ilkley, UK) was advocated for up to four hours a day in custom made trays, for two to four weeks prior to any further treatment. Children (with parental input) who were not satisfied with the aesthetic outcome at their one-month review were given the option of repeating the resin infiltration procedure or having a direct resin composite restoration (Filtek^TM^, 3M ESPE, Bracknell, UK) to further mask the enamel opacity (Figure 2). All children were treated using rubber dam and appropriate protection of soft tissues. They were advised not to have any highly coloured food or drinks for four days following their treatment.

## 3. Results

### 3.1. Participants

A total of 111 children who met the inclusion criteria were initially approached to participate in the study. Of these, 103 (92.8% response rate) agreed to take part and signed their consent forms. However, there was a drop out of 10 patients, with a final 93 children (90.3% completion rate) completing their treatment and pre- and post-treatment questionnaires. The characteristics of these 93 participants are summarised in Table 1. The mean age was 10.95 years (SD = 2.54; range = 7–16), with a higher proportion of girls (59.1%, n = 55) than boys. There was representation from ethnic minority groups as well as from children from the most socially deprived quintiles. Indeed, around 40% of participants were from the two most deprived quintiles. For completeness, Table 1 also provides details of the treatment regimens employed for these participants, where it can be seen that the majority underwent microabrasion immediately followed by resin infiltration (n = 66.71%). Only two children/parents requested resin composite restorations to achieve optimum aesthetics following one or more of the other treatment options.

### 3.2. Oral Health-Related Quality of Life 

Data from the 93 child-completed C-OHIP-SF19 questionnaires are presented in Table 2, at both baseline and one-month review. No questionnaire had any missing data as clinicians (N.H. and J.L.) were able to check that they had been fully completed at the time of issue. 

The mean C-OHIP-SF19 score at baseline (before treatment) was 47.00 (SD = 9.29; range = 0–76), and this increased significantly following treatment at the one-month review to 58.24 (SD = 9.42; range = 0–76) (*p* < 0.001). This was a large effect size of 11.24 points (14.7% of the total possible score), which suggested that children perceived themselves as having a marked improvement in their overall OHRQoL. In terms of floor and ceiling effects, no children gave the lowest possible score (zero) at T_0_ and T_1_ and one child gave the highest possible score (76) at T_0_ and none at T_1_. C-OHIP-SF19 reliability was assessed through a Cronbach’s alpha coefficient of the items of the scale. The Cronbach’s alpha coefficient of C-OHIP-SF19 at T_0_ and T_1_ were 0.748 and 0.810, respectively, indicating very good internal consistency [26].

Furthermore, mean scores for each dimension within the measure were also significantly increased following the intervention, with the greatest change seen for socio-emotional wellbeing: Oral health T_0_ = 11.25 and T_1_ = 14.15 (*p* < 0.001); functional wellbeing T_0_ = 13.28 and T_1_ = 14.16 (*p* < 0.001); and socio-emotional wellbeing T_0_ = 22.46 and T_1_ = 29.92 (*p* < 0.001). Further analysis showed that the total mean scores for C-OHIP-SF-19 and all its dimensions were significantly increased at one-month follow-up for both age groups (7–10 years and 11–16 years). 

There were no significant differences in total C-OHIP-SF19 or domain scores according to gender either at baseline or one-month review (*p* > 0.05, Mann-Whitney U test). Data are shown separately for male and female participants and according to age groups (7–10 years and 11–16 years) for information (Table 2).

### 3.3. Attendance and Treatment

The purpose of this research was to evaluate patient-reported outcomes rather than to evaluate the outcomes of the different interventions from a clinical (objective) perspective. However, for completeness, Table 1 provides the details of the different treatment regimens employed. It can be noted that almost a third of children (31.2%, n = 29) (data not tabulated) had a second course of treatment, suggesting they were not completely satisfied with the initial clinical outcome. There were no adverse outcomes, such as loss of tooth vitality or receipt of a patient complaint, throughout the programme of research.

## 4. Discussion

To the authors’ knowledge, this is the first study to show a change (improvement) in children’s self-reported OHRQoL following a clinical intervention to reduce the visibility of incisor opacities associated with MIH. Previous data, highlighting the negative impacts of MIH on OHRQoL, have largely been derived from cross-sectional school-based populations [12,27]. Clinical-based investigations in young children, with any type of enamel defect, are sparse [2,18,19]. However, a recent randomised clinical trial did demonstrate improved OHRQoL following microabrasion/tooth whitening for young Brazilian adults with dental fluorosis [28]. Although not a universal finding, some OHRQoL studies have shown gender differences, with girls reporting lower OHRQoL than boys, in relation to a variety of dental conditions [29,30]. On first inspection, therefore, it was surprising that the current investigation did not reveal any significant gender differences in reported OHRQoL, either before or after aesthetic treatment. However, it must be noted that there was a greater proportion of females (59.1%) than males in this study group, suggesting that girls (and their parents) may have been more likely to seek specialist treatment (hospital referral) in the first place, potentially masking any true gender differences. 

### 4.1. Study Strengths

The ease of participant recruitment was a key facilitator in the success of this study, ensuring that the required sample size was readily met. Indeed, response and completion rates of 92.8% and 90.3%, respectively, are considered exceptional in the authors’ experience as a response rate of 50–60% is more the norm in their unit [29,31]. It can only be assumed that children with MIH, together with their families, are highly motivated to seek ‘aesthetic’ dental treatment, demonstrated by their excellent attendance rates and willingness to partake in research. It should be noted, however, that children were offered the same treatment, irrespective of whether or not they wished to take part in the study, thus there was no incentive for study participation. It was encouraging that there was representation from ethnic minority children as well as those from areas of high deprivation, allowing the findings to be widely generalisable. However, it is acknowledged that the proportion of participants from an ethnic minority group (8.6%) was slightly lower than the UK’s ethnic minority group as a whole (14%) [32].

### 4.2. Limitations

It was important to select the most appropriate OHRQoL measure for the purposes of this study, having reviewed the acknowledged advantages and disadvantaged of the most widely used and validated instruments [24]. One factor, prompting the choice of C-OHIP-SF19, was its inclusion of a specific item referring to ‘marks’ on teeth, making it highly relevant to the proposed research. However, the response format, which asks children to state how often in the past three months they have been affected by the marks or discolouration, may not actually reflect how children describe the impact. The authors’ impression is that children are much more likely to quantify the impact of their dental condition in terms of its severity (how much or little) rather than the frequency of that impact [33]. However, in the absence of an alternative instrument, C-OHIP-SF19 was considered fit for purpose. The fact that it was sensitive to change, following a dental intervention, with limited ceiling and floor effects, further supported its application in this study.

In addition to their affected incisors, participants in this study also had a variable number of hypomineralised first permanent molars. Although these were unlikely to be of cosmetic concern, they undoubtedly presented other impacts for children, in terms of treatment burden and dental sensitivity [34]. One of the inclusion criteria for participants was that their first permanent molars were considered ‘asymptomatic’, having been previously extracted or restored. However, it cannot be assumed that the status of the child’s first permanent molars did not influence their overall OHRQoL. The potential confounding effect of the severity of the child’s MIH as a whole will therefore need to be considered in future analysis. It is also noted that this study did not include a control group. Withholding ‘cosmetic’ treatment for children with MIH would be unethical, however, the study could have included a control group where treatment provision was ‘delayed’ for two months. It would also be interesting to compare self-report OHRQoL in children with incisor opacities, but who do not express a wish for any aesthetic intervention. This would have allowed the investigators to verify that OHRQoL remained stable in the control group over this time period and change in the experimental group was due to the dental intervention itself.

An acknowledged limitation of quantitative research, such as this, is that it fails to capture novel or deeper insights. A numerical change in mean reported C-OHIP-SF19 score, or indeed individual domains following the dental intervention, does not fully explain how and why children feel better about themselves. To gain a greater understanding of how children perceive their oral health following masking of their incisor opacities, a qualitative approach would be warranted. This type of enquiry could also illuminate patient and parent treatment expectations and experiences, thereby helping providers to evaluate and improve their overall quality of care. A previous study in the authors’ unit, which sought children’s satisfaction following aesthetic treatment for a variety of enamel defects, revealed that expectations were not always met, with some young patients reportedly disappointed that the treatment outcome was not ‘perfect’ [19]. 

### 4.3. Clinical Implications

This research focused on the patient’s perspective of treatment, by looking at change in self-reported OHRQoL following a simple intervention. Although children’s involvement in oral health research is strongly advocated [35], this study will undoubtedly raise questions about clinical outcomes. There is a paucity of evidence to support the use of one clinical regimen over another in the management of incisor opacities. Indeed, a recent systematic review on treatment modalities for children with MIH concluded that ‘no recommendations can be given for MIH-incisors’ [34]. As such, the predictability and overall success of minimally invasive techniques in reducing the visibility of incisor opacities remains uncertain. Clinicians are therefore likely to adopt a variety of techniques, drawing on their own experiences and preferences. However, with the advent of newer techniques, such as resin infiltration, there is a clear need for clinical trials to better inform decision-making.

### 4.4. Future Research 

As indicated above, future MIH research needs to consider both patient-reported outcomes as well as clinical outcomes. Although it should be borne in mind that there is rarely a direct correlation between biomedical measures and patient-reported measures [1]. The present study is, however, ongoing and will determine patient-reported and clinical outcomes at six-months, providing valuable and unprecedented longitudinal data. Other factors that may theoretically predict OHRQoL, as a primary outcome measure, could include a variety of patient characteristics (ethnicity, deprivation, age, sense-of-self) and clinical characteristics (caries experience, MIH severity, orthodontic status), and these could be considered within an appropriate theoretical model and more complex statistical analysis. It is also vital that future laboratory and clinical research attempts to relate clinical outcomes (change in opacity colour, visibility, and size) with both initial lesion characteristics and the treatment regimen undertaken. This is a challenging and exciting area of research, which offers great scope to improve children’s oral health and treatment experiences.

## 5. Conclusions

Accepting the limitations of an uncontrolled study design, this clinical research has provided persuasive and novel evidence for improvement in self-reported OHRQoL following minimally invasive aesthetic treatment for masking the visibility of incisor opacities in children with MIH.

## Figures and Tables

**Table 1:** Sociodemographic and clinical characteristics of study participants (n = 93).

Patient Variable		n (%) Mean (SD, Range)
Age (years)	All Participants	10.95 (2.54, 7–16)
	7–10	50 (53.8)
	11–16	43 (46.2)
Gender	Male	38 (40.9)
	Female	55 (59.1)
Ethnic background	White British/Northern European	85 (91.4)
	Any other group	8 (8.6)
Deprivation score	1st Quintile (least deprived)	18 (19.4)
	2nd Quintile	24 (25.8)
	3rd Quintile	14 (15.1)
	4th Quintile	14 (15.1)
	5th Quintile (most deprived)	23 (24.7)
Number of treated teeth		3.1 (2.65, 1–12)
Treatment regimen	Microabrasion alone	6 (6.5)
	ICON alone	4 (4.3)
	Microabrasion followed by ICON	66 (71.0)
	Microabrasion followed by tooth whitening	8 (8.6)
	Microbrasion and/or ICON followed by resin composite restoration	2 (2.2)
	Tooth whitening followed by microabrasion and/or ICON	7 (7.5)

**Table 2:** Mean (SD) Child Oral Health Impact Profile (C-OHIP-SF19) scores before treatment (baseline) and one-month following treatment and according to gender.

C-OHIP-SF19 Participants		Pre-Treatment C-OHIP-SF19 Mean (SD, Range)	Post-Treatment C-OHIP-SF19 Mean (SD, Range)	Change in C-OHIP-SF19 Score	Significance (*p*) Wilcoxon Signed Rank Test
Overall score	All participants (n = 93)	47.00 (9.29, 0–76)	58.24 (9.42, 0–76)	11.24	*p* < 0.001
	Female (n = 55)	46.15 (9.48, 0–76)	58.51 (9.77, 0–76)	12.36	*p* < 0.001
	Male (n = 38)	48.24 (8.99, 0–76)	57.84 (9.01, 0–76)	9.60	*p* < 0.001
	Age 7–10 (n = 50)	47.06 (8.89, 0–76)	59.66 (9.38, 0–76)	12.60	*p* < 0.001
	Age 11–16 (n = 43)	46.93 (9.85, 0–76)	56.58 (9.30, 0–76)	9.65	*p* < 0.001
Oral health domain	All participants (n = 93)	11.26 (2.78, 0–20)	14.15 (3.34, 0–20)	2.89	*p* < 0.001
	Female (n = 55)	11.13 (2.93, 0–20)	14.55 (3.42, 0–20)	3.42	*p* < 0.001
	Male (n = 38)	11.45 (2.58, 0–20)	13.58 (3.32, 0–20)	2.13	*p* < 0.001
	Age 7–10 (n = 50)	10.58 (2.85, 0–20)	14.06 (3.34, 0–20)	3.48	*p* < 0.001
	Age 11–16 (n = 43)	12.05 (2.51, 0–20)	14.26 (3.50, 0–20)	2.21	*p* < 0.001
Functional wellbeing domain	All participants (n = 93)	13.28 (2.59, 0–16)	14.16 (1.87, 0–16)	0.88	*p* < 0.001
	Female (n = 55)	13.35 (2.25, 0–16)	14.20 (1.86, 0–16)	0.85	*p* < 0.011
	Male (n = 38)	13.18 (3.04, 0–16)	14.10 (1.93, 0–16)	0.92	*p* < 0.030
	Age 7–10 (n = 50)	13.20 (2.24, 0–16)	13.98 (1.85, 0–16)	0.78	*p* < 0.016
	Age 11–16 (n = 43)	13.37 (2.97, 0–16)	14.37 (1.90, 0–16)	1.00	*p* < 0.020
Socio-emotional wellbeing domain	All participants (n = 93)	22.46 (7.67, 0–40)	29.92 (6.42, 0–40)	7.46	*p* < 0.001
	Female (n = 55)	21.67 (8.22, 0–40)	29.76 (6.70, 0–40)	8.09	*p* < 0.001
	Male (n = 38)	23.60 (6.81, 0–40)	30.15 (6.05, 0–40)	6.55	*p* < 0.001
	Age 7–10 (n = 50)	23.28 (7.76, 0–40)	31.62 (6.06, 0–40)	8.34	*p* < 0.001
	Age 11–16 (n = 43)	21.51 (7.61, 0–40)	27.95 (6.32, 0–40)	6.44	*p* < 0.001
